# Synthesis of LiTiO_2_ Nanocrystals/Ordered Mesoporous Carbon Composite Hosts for High‐Performance Lithium–Sulfur Batteries

**DOI:** 10.1002/smsc.202300019

**Published:** 2023-04-12

**Authors:** Changyao Wang, Wei Zhang, Mengmeng Liu, Linlin Duan, Bing Ma, Xingmiao Zhang, Wei Li

**Affiliations:** ^1^ Department of Chemistry Laboratory of Advanced Materials Shanghai Key Lab of Molecular Catalysis and Innovative Materials iChEM and State Key Laboratory of Molecular Engineering of Polymers Fudan University Shanghai 200433 China

**Keywords:** coassemblies, composites, Li–S batteries, mesoporous materials

## Abstract

Metal oxide nanocrystals/mesoporous carbon composite materials are promising in the energy storage field. However, the construction of stoichiometric ternary nanocrystals‐functionalized mesoporous carbon materials remains a great challenge. Herein, the synthesis of ultradispersed and ultrasmall LiTiO_2_ nanocrystals/ordered mesoporous carbon composites via a chelation‐mediated multicomponent coassembly strategy is reported. In this case, the self‐assembly into ordered mesostructures and the crystallization of nanoparticle processes can be decoupled by the molecular chelate strategy where citrate ligands can effectively inhibit the hydrolysis and phase separation of metal oxide precursors and confine the crystallization into nanocrystals without aggregation. The obtained 33%‐LiTiO_2_–OMC composites present a high specific surface area (≈912 m^2^ g^−1^), a large pore volume (≈0.62 cm^3^ g^−1^), a uniform pore size (≈4.1 nm), and ultradispersed LiTiO_2_ nanocrystals (*≈*3 nm). When loading 60% sulfur, the composites exhibit a high reversible capacity (966 mAh g^−1^ after 100 cycles at 0.5C), an excellent rate capacity (700 mAh g^−1^ at 5C), and a long‐term cycling performance (63% retention after 1000 cycles at 5C). This method is very simple and reproducible, which paves a new way for the design and synthesis of functional mesoporous materials.

## Graphical abstract

A chelation‐mediated multicomponent coassembly strategy is developed for the controllable synthesis of ultradispersed and ultrasmall LiTiO_2_ nanocrystals/ordered mesoporous carbon composites with high specific surface areas, large pore volumes, and uniform pore size. When loading 60% sulfur, the composites exhibit a high reversible capacity, an excellent rate capacity, and a long‐term cycling performance.

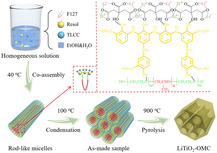

## 1 Introduction

Transition metal oxide (TMO) nanocrystals have shown great application potential in various fields such as catalysis, sensor, energy conversion, and storage.^[^ 1, 2, 3, 4, 5, 6 ^]^ However, the poor stability of TMO nanocrystals affects their performances greatly. Easy migration, aggregation, coalescence, and broken are usually observed, accompanied with drastic performance degeneration.^[^ 7, 8, 9 ^]^ Integration with support materials is an effective route to solve the aforementioned disadvantages.^[^ 10, 11, 12, 13, 14, 15, 16, 17, 18 ^]^ Benefiting from the large pore sizes, high surface areas, ordered structures, and nanometer‐scale pore walls, mesoporous materials have been widely used as the support materials for TMOs nanocrystals.^[^ 19, 20, 21, 22, 23, 24, 25, 26 ^]^ The structural characteristic of mesoporous materials is beneficial for stabilizing the nanocrystals during the reaction process.

Ex situ and in situ methods are the two most popular synthesis routes to the construction of TMO nanocrystals‐functionalized mesoporous materials.^[^ 27, 28 ^]^ For the ex situ method, mesoporous materials are first synthesized and then metal oxide precursors are generally introduced into the mesopores through the wetness impregnation technique. After post‐treatments, the TMO nanocrystals are formed in the mesoporous matrix.^[^ 29, 30, 31, 32 ^]^ However, this method is tedious, time‐consuming, and costly. Besides, the TMO nanocrystals are usually located in the mesochannels with uncontrollable size and spatial distribution, which also lead to easy migration, aggregation, and breakage under the working condition. On the contrary, the in situ coassembly synthesis method exhibits great promise to the synthesis of TMO nanocrystals‐functionalized mesoporous materials with highly uniform spatial and size distribution. Meanwhile, the nanocrystals are embedded in the frameworks which are beneficial for its stability. In this method, the surfactants coassemble with matrix and metal oxide precursors into ordered mesostructures first. Then, the nanocrystals can be formed in the pore walls uniformly after thermal treatment for conversion and template removal. Nevertheless, the compositions of incorporated metal oxides nanocrystals are still limited to single components such as TiO_2_, WO_3_, CoO_*x*_, and Fe_2_O_3_.^[^ 33, 34, 35, 36 ^]^ Until now, the synthesis of stoichiometric ternary nanocrystals metal oxide nanocrystals‐functionalized mesoporous materials with ultradispersity feature is difficult due to the following reasons. First, the unmatched and complex hydrolysis condition of different metal precursors usually lead to the macroscopic phase separation in the coassembly process. Second, the ununiform dispersity of each metal precursor in the frameworks results in the respective crystallization of each metal oxide. As a result, the formation of pure ternary metal oxide nanocrystals in the frameworks is difficult. Finally, high temperature is needed for the crystallization of ternary metal oxides nanocrystals, leading to large and ununiform particle size and uncontrollable spatial distribution. How to control the coassembly and subsequent crystallization process is still a great challenge in the synthesis of stoichiometric multimetal oxide nanocrystals‐functionalized mesoporous materials.^[^ 37, 38, 39, 40, 41 ^]^

Herein, we developed a chelation‐mediated multicomponent coassembly strategy for the controllable construction of ternary LiTiO_2_ nanocrystals/ordered mesoporous carbon (denoted as *X*‐LiTiO_2_‐OMC, wherein *X* represents the LiTiO_2_ percentage in the composites) composites. The Ti^4+^/Li^+^/citrate chelate (TLCC; Ti^4+^:Li^+^ = 1:1) is constructed as novel metal precursor in which the carboxyl groups coordinate Li^+^ and Ti^4+^ homogeneously through strong electrostatic interaction, which is beneficial to the dispersity of metal ions in the matrix. Meanwhile, the citrate chelate can interact with PEO segments and resol molecular through OH groups, which promotes the formation of ordered mesostructures without macroscopic phase separation. Furthermore, the excellent dispersity of metal ions ensures the formation of ultradispersed and ultrasmall stoichiometric LiTiO_2_ nanocrystals in the mesoporous carbon matrix after pyrolysis. As a result, the obtained LiTiO_2_–OMC composites display a high specific surface area (≈912 m^2^ g^−1^), a large pore volume (≈0.6 cm^3^ g^−1^), a uniform pore size (≈4.1 nm), and uniformly dispersed LiTiO_2_ nanocrystals (≈3 nm). When being used as the host for sulfur, the composites cathode exhibits a remarkably high reversible capacity (966 mAh g^−1^ after 100 cycles at 0.5C), an excellent rate capacity (700 mAh g^−1^ at 5C), and long‐term cycling performance (63% retention after 1000 cycles at 5C).

## 2 Results and Discussion

**Figure 1:**
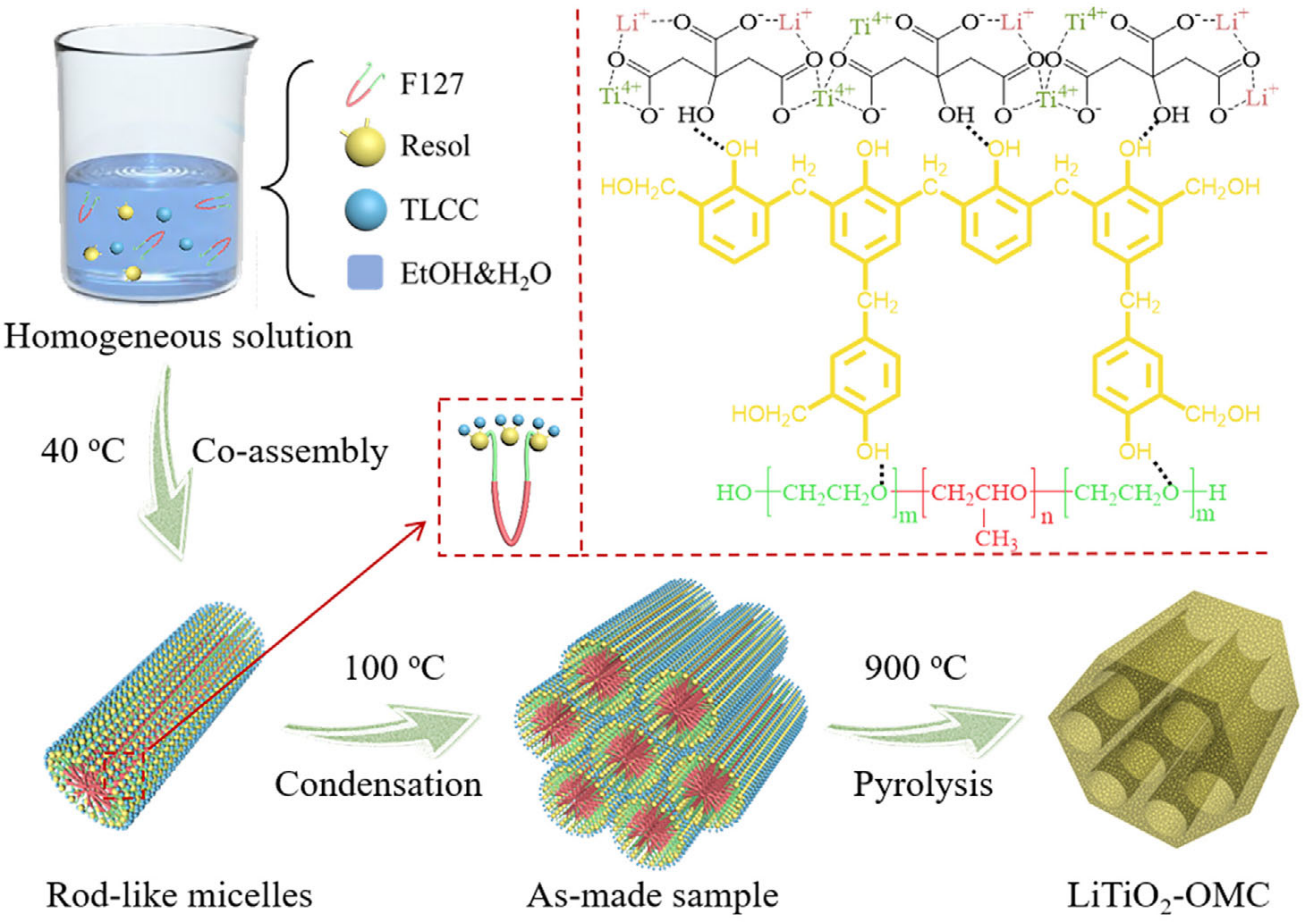
Scheme for the fabrication of LiTiO_2_–OMC via the chelation‐mediated multicomponent coassembly strategy.

The scheme for the fabrication of LiTiO_2_–OMC via the chelation‐mediated multicomponent coassembly strategy is illustrated in **Figure** **1**. First, the stoichiometric Ti^4+^/Li^+^/citrate chelate (TLCC; Ti^4+^: Li^+^ = 1:1) is synthesized as the precursor (Figure S1, Supporting Information). After that, the PEO segment of the Pluronic F127 coassemble with the resol and TLCC precursors to rod‐like micelles through hydrogen bonding interaction. The formed rod‐like micelles can be further packed into ordered mesostructures (as‐made sample) with the evaporation of solvent. Finally, the product LiTiO_2_–OMC composites can be obtained after pyrolysis at 900 °C in which the ternary LiTiO_2_ nanocrystals are uniformly distributed with an ultrasmall particle size.

**Figure 2:**
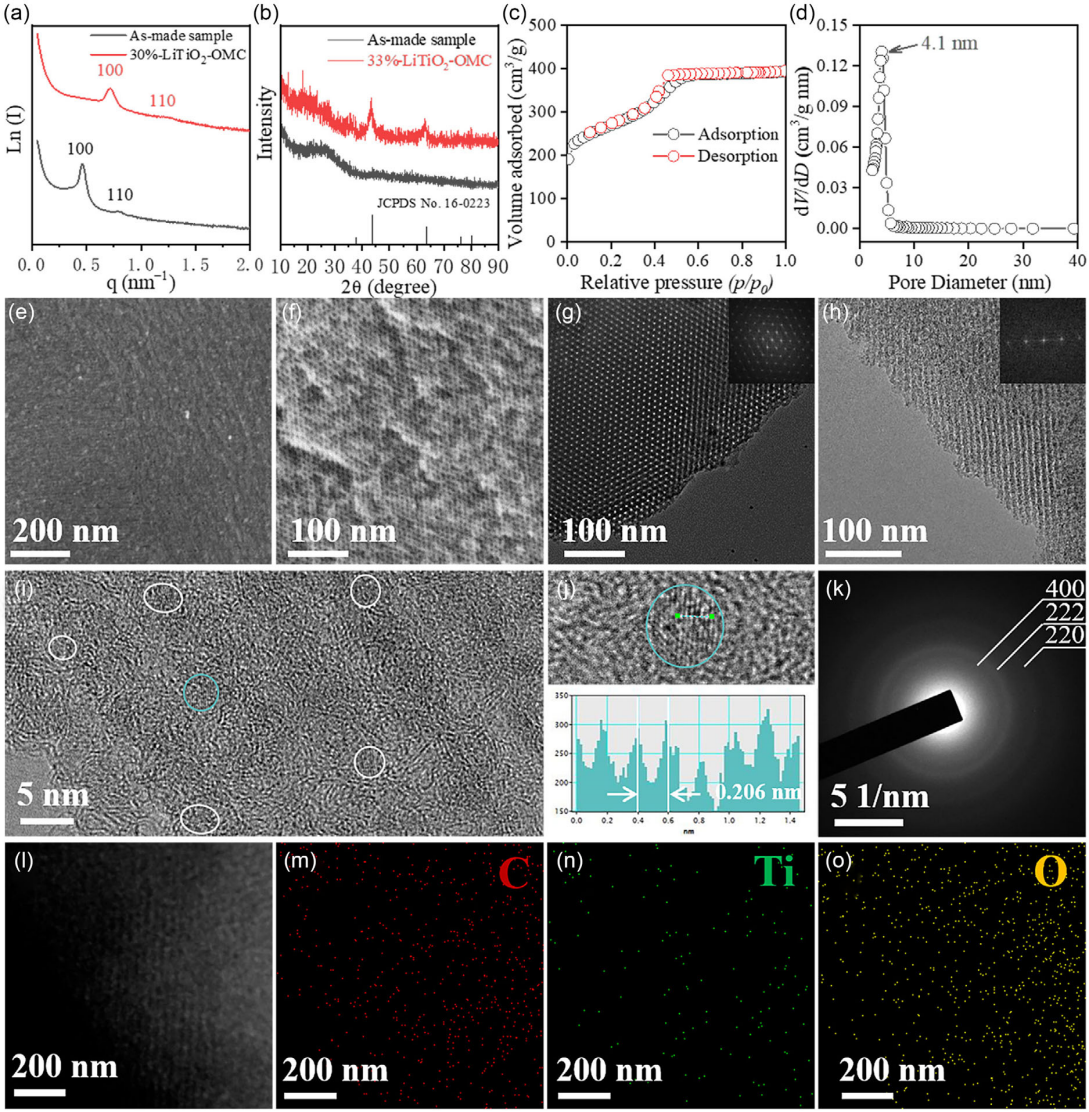
a) SAXS and b) XRD patterns of the as‐made sample and the ultradispersed and ultrasmall LiTiO_2_ nanocrystals/ordered mesoporous carbon (33%‐LiTiO_2_–OMC) composites prepared by the chelation‐mediated multicomponent coassembly strategy after pyrolysis at 900 °C in N_2_, respectively. c) Nitrogen sorption isotherms and d) the corresponding pore size distribution curve of the 33%‐LiTiO_2_–OMC composites. e,f) FESEM images: e) surface, f) cross section; g–i) TEM images taken along the g) [100], h) [110]; i) HRTEM image; j) HRTEM image and corresponding crystal lattice distance plot of a single LiTiO_2_ crystal; k) SAED pattern; l) STEM and m–o) EDS mapping of C, Ti, O elements of the 33%‐LiTiO_2_–OMC composites, respectively. The inset in (h) shows the corresponding diameter distribution histogram of the LiTiO_2_ nanocrystals.

Taking the 33%‐LiTiO_2_–OMC as an example, we explored the synthetic process carefully. Two well‐resolved scattering peaks with *q* value of 0.462 and 0.784 nm^−1^ can be observed from the small‐angle X‐ray scattering (SAXS) pattern (**Figure** **2**a) of the as‐made sample which can be attributed to the 100 and 110 reflections of the ordered hexagonal mesostructures. After pyrolysis at 900 °C in N_2_, two scattering peaks can be still observed but shifted to 0.714 and 1.240 nm^−1^ (Figure 2a), demonstrating the existence of drastic shrinkage (35.3%) of the ordered mesostructures. The X‐ray diffraction (XRD) pattern (Figure 2b) of the as‐made sample displays a broadening peak around 25°, attributed to the amorphous carbon frameworks. No other diffraction peaks can be observed in this XRD pattern, indicating no macroscopic phase separation during the coassembly process. After heat treatment, the XRD pattern (Figure 2b) of the 33%‐LiTiO_2_–OMC composites displays two broadening peaks at 43.8 and 63.5°, indexed to the 200 and 220 reflections of the cubic LiTiO_2_ structure with the *Fm* $\bar{3}$ *m* space group (JCPDS Card No. 16‐0223). The broadening diffraction peaks demonstrate the ultrasmall particle size of LiTiO_2_ in the frameworks and the average size is calculated to be ≈4.1 nm based on the Debye–Scherrer equation (Figure S2, Supporting Information).^[^ ^37^ ^]^

**Figure 3:**
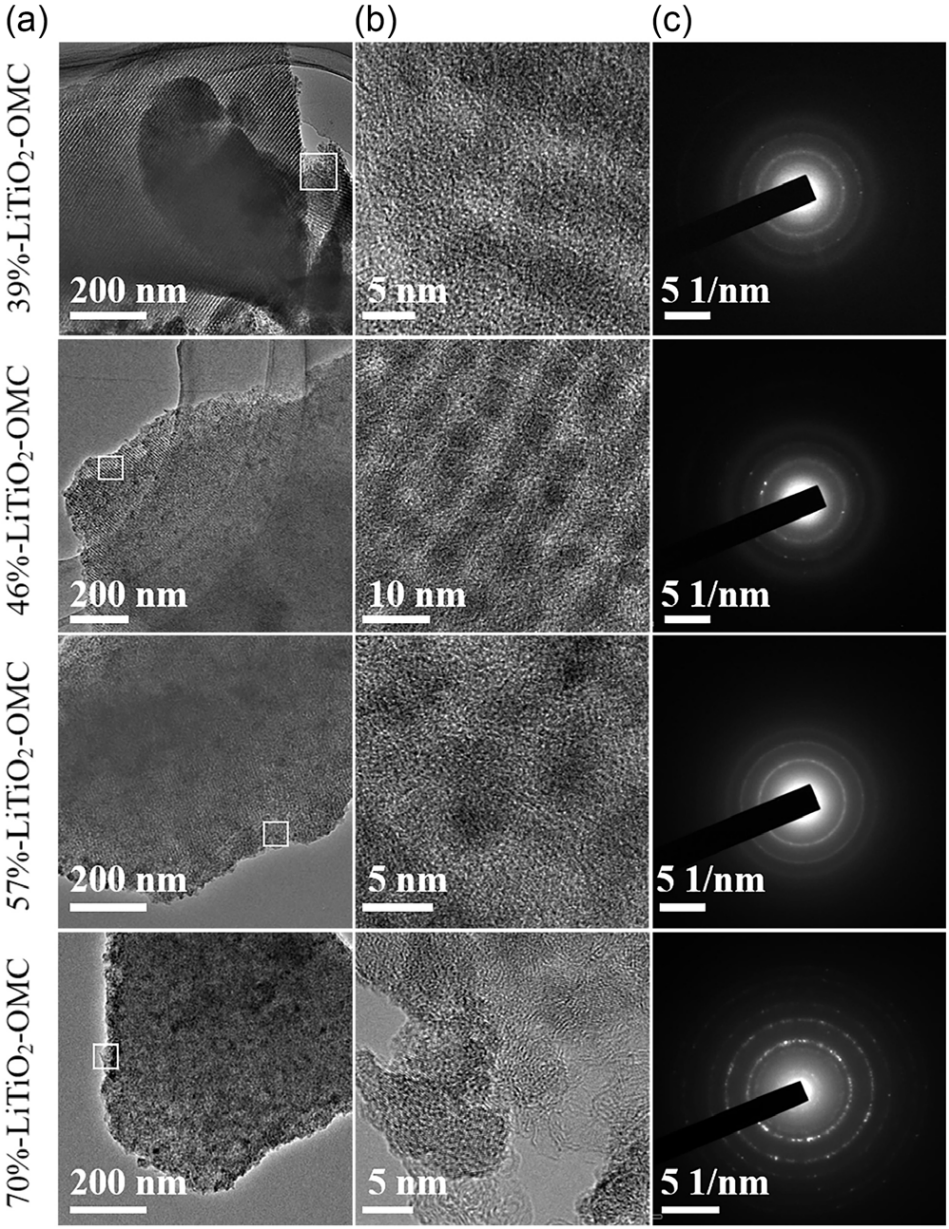
a) TEM, b) HRTEM, and c) corresponding SAED patterns of the 39%‐LiTiO_2_–OMC, 46%‐LiTiO_2_–OMC, 57%‐LiTiO_2_–OMC, and 70%‐LiTiO_2_–OMC composites prepared by the chelation‐mediated multicomponent coassembly strategy after pyrolysis at 900 °C in N_2_, respectively.

N_2_ sorption isotherms (**Figure** **3**c) of the 33%‐LiTiO_2_–OMC composites obtained after calcination at 900 °C in N_2_ display characteristic type IV curves with a H2 hysteresis loop, proving the existence of uniform mesopores. The Brunner–Emmet–Teller (BET) surface area and pore volume are calculated to be 912 m^2^ g^−1^ and 0.62 cm^3^ g^−1^, respectively. The relative low pore volume resulted from the thicker pore wall.^[^ 38, 39 ^]^ The pore size distribution curve (Figure 2d) displays that the pore size is about 4.1 nm. Two peaks at around 1344 cm^−1^ (D band) and 1596 cm^−1^ (G band) can be observed from the Raman spectrum of the 33%‐LiTiO_2_–OMC composites (Figure S3, Supporting Information). The intensity ratio of D/G bands is calculated to be 1.18, suggesting the amorphous structure of the carbon frameworks. Based on the thermogravimetric analysis (TGA) result (Figure S4, Supporting Information), the carbon content in the 33%‐LiTiO_2_–OMC is estimated to be 67%. The X‐ray photoelectron spectroscopy (XPS) survey spectrum of the 33%‐LiTiO_2_–OMC composites exhibits the peaks well corresponding to Ti, O, Li, and C elements (Figure S5a, Supporting Information). Four peaks can be detected from the high‐resolution Ti_2p_ core‐level XPS spectrum (Figure S5b, Supporting Information) at 464.3, 462.5, 458.6, and 456.8 eV, respectively, demonstrating the existence of Ti^4+^ and Ti^3+^. The existence of Ti^4+^ species can be attributed to the partial oxidation of Ti^3+^ on the surface of LiTiO_2_ nanocrystal.^[^ 40, 41 ^]^ The O_1s_ XPS spectrum displays two peaks at 532.0 and 530.1 eV due to the O–Li and O–Ti bands (Figure S5c, Supporting Information). Meanwhile, only one peak at 55.3 eV can be observed from the Li_1*s*_ XPS spectrum (Figure S5d, Supporting Information), which can be attributed to the Li^+^ species.

Field‐emission scanning electron microscopy (FESEM; Figure 2e,f) images of the 33%‐LiTiO_2_–OMC composites display the existence of the uniform mesopores on the surface and cross section. Transmission electron microscopy (TEM) images taken from the [100] and [110] directions show the existence of ordered mesochannels, in agreement with the SAXS result. The high‐resolution TEM (HRTEM) image (Figure 2i) taken from the [110] direction demonstrates that these LiTiO_2_ nanocrystals are uniformly dispersed in the amorphous carbon walls. The average particle size is estimated to be ≈3.4 nm based on the statistical results of TEM images (Figure S6, Supporting Information), in agreement with the result of XRD pattern, confirming the ultradispersity and ultrasmall features of LiTiO_2_ nanocrystals. In addition, the mesopore size is estimated to be around 3.9 nm, consistent with the N_2_ sorption result. The HRTEM image of a single nanocrystal clearly shows that the lattice space is ≈0.206 nm, corresponding to the (200) plane of the LiTiO_2_ crystal phase (Figure 2j). Meanwhile, the selected‐area electron diffraction (SAED) pattern (Figure 2k) reveals several spotty diffraction rings, further demonstrating the polycrystalline nature. Besides, the high‐angle annular dark‐field scanning TEM (HAADF‐STEM) image (Figure 2l) also displays the ordered mesostructures and the uniformly dispersed LiTiO_2_ nanocrystals in the pore walls. The corresponding elemental mapping images (Figure 2m–o) and energy‐dispersive X‐ray spectroscopy (EDX) (Figure S7, Supporting Information) clearly demonstrate that these Ti, O, and C elements are distributed on the whole frameworks uniformly.

The content of LiTiO_2_ nanocrystals in the LiTiO_2_–OMC composites (denoted as *X*‐LiTiO_2_–OMC, wherein *X* represents the LiTiO_2_ percentage in the composites (Figure S8, Supporting Information)) can be tuned facilely by adjusting the mass ratio of resol/TLCC precursors and their structural properties are listed in the Table S1, Supporting Information. SAXS patterns (Figure S9, Supporting Information) of the as‐made samples show that the highly ordered mesostructures can be retained under different resol/TLCC ratios. TEM images (Figure 3a) display that the mesopores can be retained but tend to disorder with the increase in LiTiO_2_ content. That is due to the increased crystal size with content, thus destroying the regular structures partially (Figure 3b). That can be further demonstrated by the increased diffraction intensity of the SAED and XRD patterns (Figure 3c, S10, Supporting Information). It should be mentioned that the average size of the LiTiO_2_ nanocrystals is still limited to nanoscales even in the sample with high LiTiO_2_ percent, which can be attributed to the nanoconfinement effect of carbon wall. Besides, the surface areas, pore volumes, and pore size decrease with the increased LiTiO_2_ content (Figure S11, Supporting Information).

When directly using titanium isopropoxide (TIPO) and LiNO_3_ as the metal precursors and resol as the carbon precursor, the resultant product (28%‐TiO_2_/lithium titanate‐functionalized mesoporous carbon) can be obtained. The TGA curve (Figure S12, Supporting Information) shows that the carbon content is about 72% in the product obtained after pyrolysis at 900 °C in N_2_. SAXS patterns (Figure S13a, Supporting Information) show that the ordered mesostructures are retained after pyrolysis. However, TEM images (Figure S14a,b, Supporting Information) of the 28%‐TiO_2_/lithium titanate composites display that hollow nanoparticles with large particle sizes (*≈*200 nm) are randomly dispersed in the ordered mesostructures. The corresponding SAED pattern (Figure S14b, Supporting Information) of the hollow nanoparticle shows the characteristic diffraction rings of mixed crystal phases with high crystallinity. HRTEM images and corresponding crystal lattice distance plots (Figure S14d–i, Supporting Information) taken from different parts of the hollow particle show the existence of impurity Li_4_Ti_5_O_12_ and TiO_2_ phases in the host LiTiO_2_ phase with large particle size, in agreement with the XRD result (Figure S13b, Supporting Information). The existence of TiO_2_/lithium titanate hollow nanoparticles can be attributed to the inhomogeneous dispersion of metal precursors in the composites due to the weak interaction force between resol and metal ions. In contrast, the abundant carboxyl groups in citrate acid are beneficial to the homogeneous dispersion of metal ions in the matrix through strong electrostatic interaction and then avoid the occurrence of such phenomenon. Meanwhile, no obvious diffraction peaks attributed to the metal precursors can be observed from the XRD pattern (Figure S14b, Supporting Information) of the as‐made sample, demonstrating that no macroscopic phase separation occurs in the coassembly process. As a contrast, the macroscopic phase separation occurs during the coassembly process when increasing the ratio of TIPO/LiNO_3_ precursors, which can be demonstrated by the weak and broadening peak in the SAXS pattern and the characteristic diffraction peak of metal precursor in the XRD pattern of the as‐made sample (Figure S15, Supporting Information). As a result, bulk LiTiO_2_ particles (Figure S16, Supporting Information) without mesostructures can be observed after pyrolysis at 900 °C in N_2_.

Based on the results mentioned earlier, we propose that the chelation‐mediated multicomponent coassembly strategy plays a vital role in the synthesis of LiTiO_2_–OMC composites. First, the carboxyl groups can coordinate Li^+^ and Ti^4+^ homogeneously through strong electrostatic interaction, which is beneficial to the dispersity of metal ions in the matrix. Besides, the hydroxyl in the citrate can interact with surfactants and resol through hydrogen bonding force. As a result, the Ti^4+^ and Li^+^ ions are dispersed in the frameworks homogeneously at the atomic scale without the macroscopic phase during the coassembly process. Meanwhile, the excellent dispersion is beneficial for the formation of pure ternary LiTiO_2_ nanocrystals with ultradispersity and ultrasmall size in the carbon frameworks uniformly. Second, the citrate can be carbonized to amorphous carbon in situ during the pyrolysis process which can confine the growth of LiTiO_2_ nanocrystal to nanoscale, which contributes to the dispersity.

**Figure 4:**
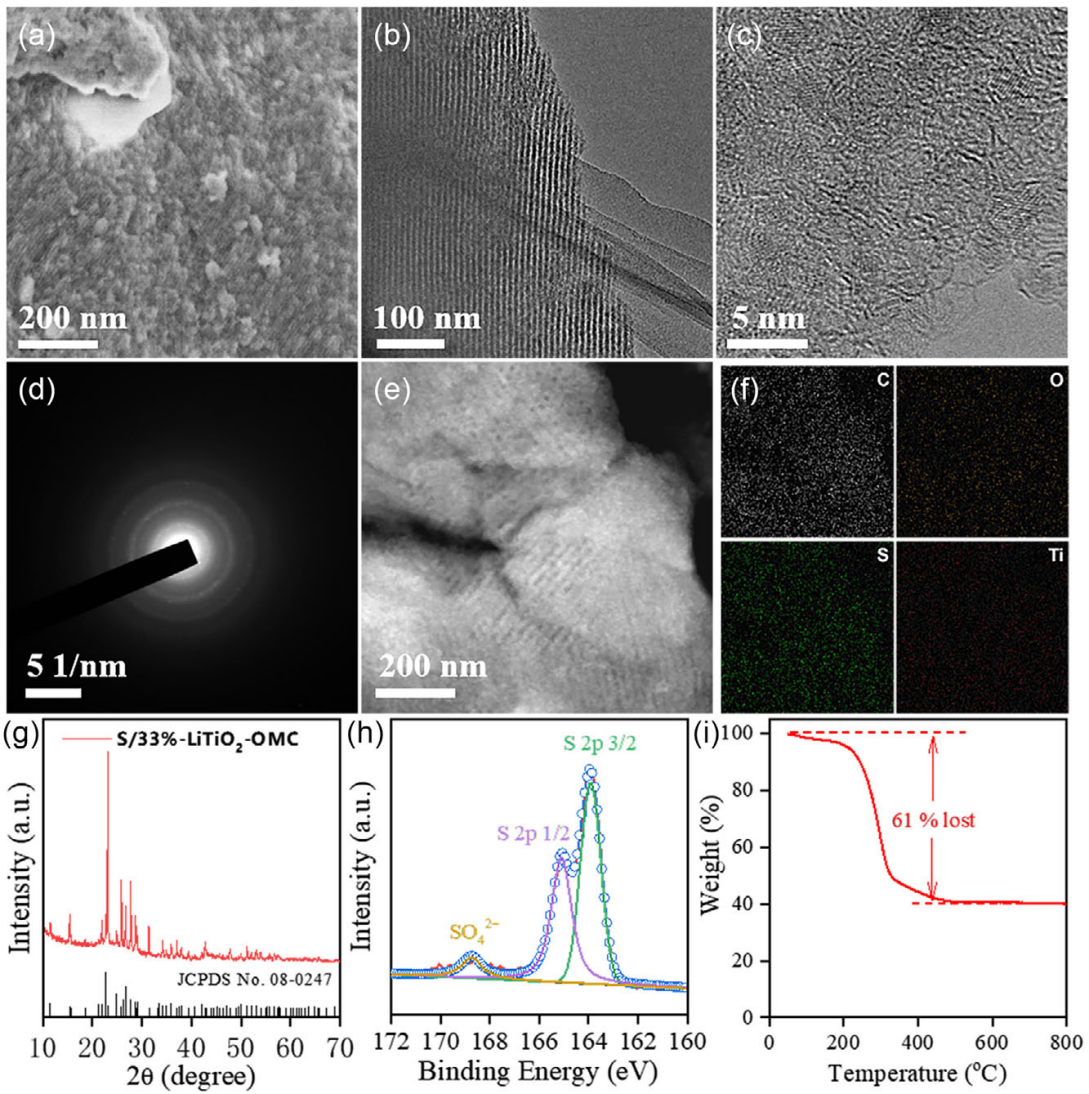
a) SEM image; b) TEM images; c) HRTEM images and d) corresponding SAED pattern; e) STEM image; f) EDS mapping of C, O, S, and Ti elements; g) XRD pattern; h) high‐resolution XPS spectrum of S and i) TGA curve of the S/33%‐LiTiO_2_–OMC composites, respectively.

To test the performances of 33%‐LiTiO_2_–OMC composites as the S host, the S/33%‐LiTiO_2_–OMC composites electrode is constructed via a molten diffusion method.^[^ ^42^ ^]^ The SEM and TEM images of the composites (**Figure** **4**a,b) display that the highly ordered mesostructures are well retained. Besides, the particle size of the LiTiO_2_ nanocrystals is still limited to around 3 nm with excellent dispersity in the carbon frameworks (Figure 4c). The SAED pattern of the composites (Figure 3d) further show the characteristic spotty diffraction rings of pure polycrystalline LiTiO_2_ without impurity crystalline phase. These results demonstrate that the molten diffusion process does not affect the structures of the 33%‐LiTiO_2_–OMC composites host. The XRD pattern (Figure 4g) of S/LiTiO_2_–OMC composites show several sharp characteristic peaks attributed to the S elementary substance, demonstrating the successful loading of S. The STEM image and corresponding elemental mapping (Figure 4e,f) further confirm the homogeneous distribution of S in the mesoporous carbon matrix.^[^ ^43^ ^]^ Besides, the N_2_ sorption isotherms (Figure S17, Supporting Information) display that the surface area and pore volume are decreased to 63 m^2^ g^−1^ and 0.1 cm^3^ g^−1^ after S infiltration. The high‐resolution S_2p_ core‐level XPS spectrum (Figure 4h) of the composites can be divided into three peaks at 168.7, 165.1, and 163.9 eV, attributed to sulfate, S_2*p*1/2_, and S_2*p*3/2_, respectively. The value of the S 2p_3/2_ peak is much lower than the pure S power (164.05 eV), attributed to the existence of LiTiO_2_ nanocrystals which can immobilize S species through forming metal—S bonds. TGA curve indicates that the S content is ≈61% (Figure 4i), which is identical with the theoretical value.

**Figure 5:**
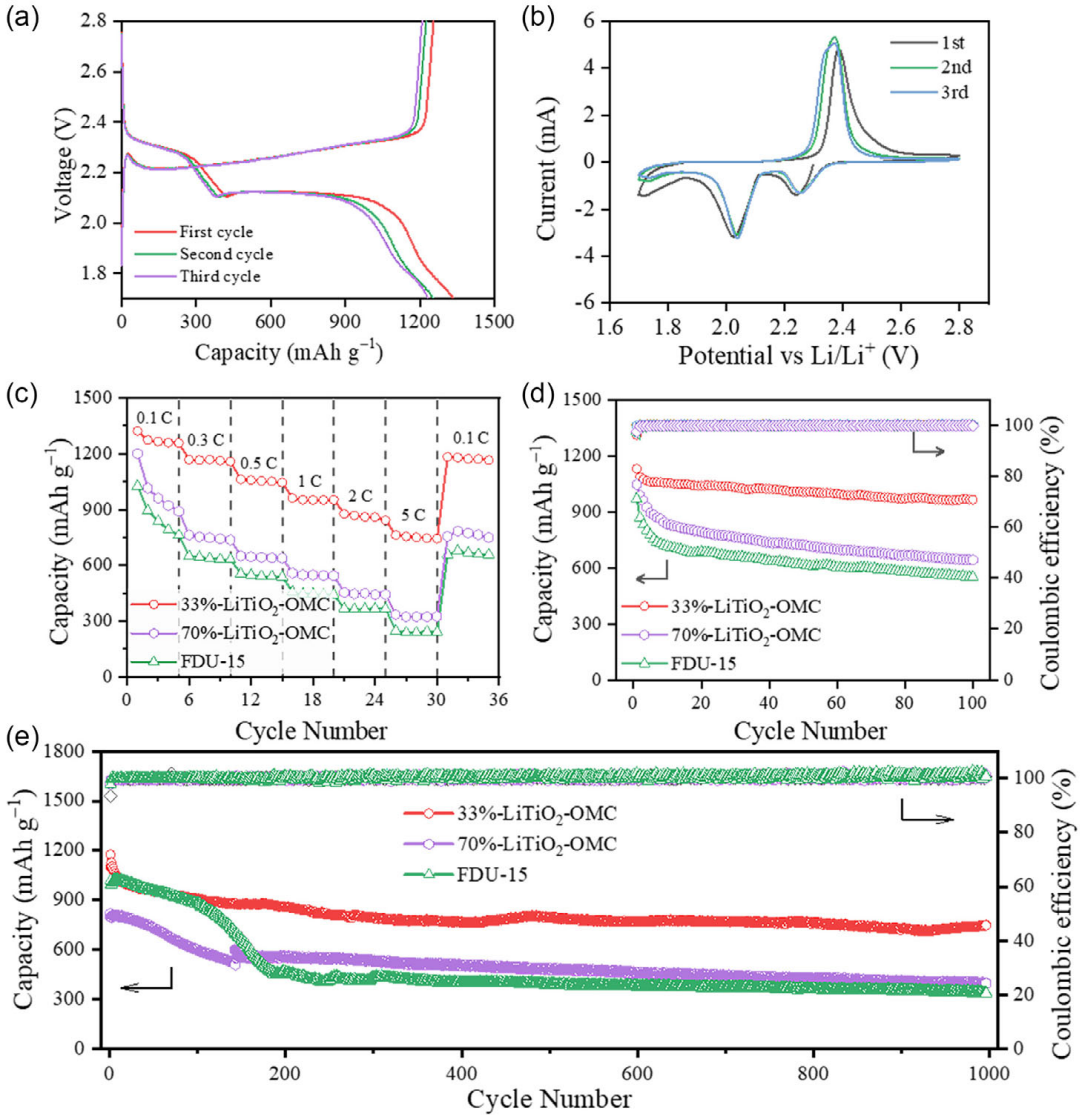
a) CV curves of the S/33%‐LiTiO_2_–OMC composites for the first three cycles at a scan rate of 0.1 mV s^−1^. b) Charge/discharge voltage profiles of the S/LiTiO_2_–OMC composites for the first, second, and third cycles at 0.1C. c) Rate performance of the S/LiTiO_2_–OMC composites, 70%‐S/LiTiO_2_–OMC composites, and S/FDU‐15 at different current densities. d) Cycling performance of the S/LiTiO_2_–OMC composites, 70%‐S/LiTiO_2_–OMC composites, and S/FDU‐15 at 0.5C and the corresponding Columbic efficiency. e) Long‐term cycling performance of the S/LiTiO_2_–OMC composites, 70%‐S/LiTiO_2_–OMC composites, and S/FDU‐15 at 2.0C and the corresponding Columbic efficiency.

The galvanostatic charge/discharge curves of the first, second, and third cycles of the S/LiTiO_2_–OMC composites electrodes are recorded at 0.1C between 1.7 and 2.8 V (**Figure** **5**a). Two typical discharge plateaus can be observed at ≈2.3 and 2.1 V, which can be attributed to the transition reaction from S_8_ molecule to soluble lithium polysulfides (LiPs, Li_2_S_*n*_, 4 ≤ *n* ≤ 8) and the subsequent reduction reaction of Li_2_S_4_ to solid‐state Li_2_S_2_ and Li_2_S, respectively.^[^ ^44^ ^]^ A high initial specific capacity of 1334.5 mAh g^−1^ can be observed from the discharge curve. Meanwhile, the initial Coulombic efficiency is as high as 94%, demonstrating the excellent immobilization effect of soluble Li_2_S_*n*_ by the LiTiO_2_ nanocrystals. The discharge specific capacity and Coulombic efficiency can be stabilized to around 1230 mAh g^−1^ and 100% from the second cycle. Cyclic voltammetry (CV) curves of S/LiTiO_2_–OMC composites electrodes are recorded at a scan rate of 0.1 mV s^−1^ (Figure 5b). Two representative reductive peaks at around 2.25 and 2.03 V can be detected during the cathodic reduction process from the CV curves, in agreement with the galvanostatic charge/discharge results. In addition, a well‐defined oxidation peak at ≈2.4 V can be observed due to the transition from Li_2_S/Li_2_S_2_ to LiPs or S_8_.

The S/33%‐LiTiO_2_–OMC composites electrode also shows excellent rate performance (Figure 5c) with the average capacities of 1260, 1069, 954, 861, 775, and 700 mAh g^−1^ at current densities of 0.1, 0.3, 0.5, 1, 2, and 5C, respectively. High specific capacity can be still observed at 5C and quickly recovered to 1142 mAh g^−1^ when the current density turns back to 0.1C. As a contrast, the S/70%‐LiTiO_2_–OMC composites and S/FDU‐15 are constructed. Drastic specific capacity loss can be observed with the increased current density. Only 324 and 243 mAh g^−1^ can be observed at 5C for S/70%‐LiTiO_2_–OMC and S/FDU‐15, respectively. Then, the cycling stability performances of three samples are evaluated at 0.5 C (Figure 5d). The Coulombic efficiency of the S/LiTiO_2_–OMC is as high as 96.2% for the first cycle and then stabilizes at around 100%. Meanwhile, the discharge specific capacity is 1131 mAh g^−1^ for the first cycle and can be retained at 966 mAh g^−1^ after 100 cycles (85% retention). In contrast, the S/70%‐LiTiO_2_–OMC composites and S/FDU‐15 electrodes display lower initial discharge specific capacity (1038 and 970 mAh g^−1^, respectively) and poor capacity retention (61.4 and 56.8%, respectively) after 100 cycles. These results demonstrate the outstanding cycling stability of the S/LiTiO_2_–OMC composites electrode. More impressively, the S/LiTiO_2_–OMC composites electrode shows an ultralong cycling life at a large current density of 2C with a 63% specific capacity retention after 1000 cycles (Figure 5e). As a contrast, only 48% and 31% specific capacity retention can be observed for the S/70%‐LiTiO_2_–OMC composites and S/FDU‐15 electrodes, respectively. Nyquist plots of three samples all show the existence of high‐frequency and low‐frequency regions simultaneously (Figure S18, Supporting Information). It should be noted here that the stability and capacity of 33%‐LiTiO_2_–OMC for Li–S battery are comparable with the most of other TiO_2_‐based hosts (Table S2, Supporting Information).^[^ 45, 46, 47, 48, 49, 50 ^]^ The S/LiTiO_2_–OMC composites electrode displays the smallest diameter of the semicircle in the high‐frequency region and the highest slope of the straight line in the low‐frequency regions than the other two samples. This result demonstrates that the S/LiTiO_2_–OMC composite electrode possesses the lowest charge transfer resistance (16.1 Ω) and highest lithium diffusion coefficient than others.

To demonstrate that the existence of the ultrasmall LiTiO_2_ nanocrystals in the ordered carbon frameworks can facilitate the adsorption (or decomposition) of the LiPs species, the adsorption experiments are carried out (Figure S19, Supporting Information). The yellow color fades away and finally became invisible within 4 h once the 33%‐LiTiO_2_–OMC composites are added into the Li_2_S_6_ solution, demonstrating the strong adsorption ability between LiTiO_2_ and LiPs species. As a contrast, the yellow color can be still observed after 24.0 h after the addition of 70%‐LiTiO_2_–OMC composites and FDU‐15 into the solution, proving their weak affinity to LiPs species. The corresponding ultraviolet–visible (UV) absorption spectra (Figure S20, Supporting Information) demonstrate that the liquid sample taken from the 33%‐LiTiO_2_–OMC composites added solution displays the weakest LiPs absorption peaks than other samples, further suggesting its strong adsorption or decomposition ability for LiPs species.

The excellent rate and cycling performances of the S/33%‐LiTiO_2_–OMC composites electrode can be attributed to its unique structures, including the high surface areas, large pore volumes, ordered mesochannels, as well as the existence of ultrasmall LiTiO_2_ nanocrystals in the carbon frameworks. The high surface areas and large pore volume are beneficial to the loading of S into the mesostructures with a large amount, contributing to the high capacity. Besides, the interconnected and uniform mesopores can not only facilitate the transport of electrolytes to interact with electrode material but also greatly shorten the ion diffusion distance, leading to rapid electrochemical reactions, high capacities, excellent cycling, and rate performance. The ultrasmall LiTiO_2_ nanocrystals display a strong affinity for LiPs and can convert them to short polysulfides quickly. In addition, the formed short polysulfides can be adsorbed on the surface of LiTiO_2_ nanocrystals. As a result, the polysulfides dissolution and shuttle effect can be greatly suppressed, leading to excellent cycling and rate performance.

## 3 Conclusion

We have demonstrated a chelation‐mediated multicomponent coassembly strategy for the controllable synthesis of ultradispersed and ultrasmall LiTiO_2_ nanocrystals/ordered mesoporous carbon (LiTiO_2_–OMC) composites host for high‐performance Li–S batteries. The used chelate agent can adjust the coassembly process and dispersity of metal ions in the matrix which ensures the formation of the ordered mesostructure and ultradispersity of LiTiO_2_ nanocrystals. The loading content of LiTiO_2_ can be tuned, even as high as 70 wt% without pore blocking and aggregation. The ultrasmall LiTiO_2_ nanocrystals can facilitate the adsorption (or decomposition) of the LiPs species, which can greatly suppress the polysulfides dissolution and shuttle effect. As a result, the S/33 wt%‐LiTiO_2_–OMC composites exhibit a high reversible capacity (966 mAh g^−1^ after 100 cycles at 0.5C) and an excellent rate capacity (700 mAh g^−1^ at 5C). Even after 1000 cycles 63% capacity can be well retained at 5C. This method is very simple and reproducible, which paves a new way to design and synthesize functional mesoporous materials.

## Conflict of Interest

The authors declare no conflict of interest.

## Supporting information

Supplementary Material

## Data Availability

The data that support the findings of this study are available from the corresponding author upon reasonable request.
