# Supplementary material for: Synthesis of LiTiO2 Nanocrystals/Ordered Mesoporous Carbon Composite Hosts for High‐Performance Lithium–Sulfur Batteries
Source: Small Sci. 2023 Apr 12;3(6):2300019. doi: 10.1002/smsc.202300019 (PMC11935920; doi:10.1002/smsc.202300019)
Supplement: Supplementary file 1 — Supplementary Material [file SMSC-3-2300019-s001.pdf]

Supporting Information

Synthesis of $LiTiO_2$ Nanocrystals/Ordered Mesoporous Carbon Composite Hosts for High-Performance Lithium–Sulfur Batteries

Changyao Wang,[a,†] Wei Zhang,[a,†] Mengmeng Liu,[a] Linlin Duan,[a] Bing Ma,[a] Xingmiao Zhang,[a] and Wei Li*,[a]

[a] Department of Chemistry, Laboratory of Advanced Materials, Shanghai Key Lab of Molecular Catalysis and Innovative Materials, iChEM and State Key Laboratory of Molecular Engineering of Polymers, Fudan University, Shanghai 200433, Chin

[†]These authors contributed equally to this work.

E-mail addresses: *weilichem@fudan.edu.cn;*

EXPERIMENTAL SECTION

Chemicals. Titanium isopropoxide [$Ti(OCH(CH_3)_2)_4$, TIPO, ⩾ 97%] was purchased from Fluka. Poly(ethylene oxide)-block-poly(propylene oxide)-block-poly(ethylene oxide) triblock copolymer Pluronic F127 [M_w = 12600, PEO_{106}-PPO_{70}-PEO_{106}] was purchased from Aldrich. Citric acid monohydrate ($C_6H_8O_7 \cdot H_2O$, AR), hydrogen chloride (HCl, AR), and ethanol (AR) were purchased from Sino−Pharm Chemical Reagent Co. Ltd. Lithium nitrate ($LiNO_3$, AR) was purchased from Aladdin. All the chemicals were used without further purification. Deionized water was used for all experiments.

Synthesis of Stoichiometric Ti^{4+}/Li^{+}/citrate Chelate. The stoichiometric Ti^{4+}/Li^{+}/citrate chelate was synthesized according to our previous method.[1] For a typical preparation, citric acid (21.0 g, 100 mmol) was dissolved in 100 mL of ethanol (solution A), titanium isoproxide (28.4 g, 100 mmol) was dissolved in 50 mL of ethanol (solution B), and lithium nitrate (6.9 g, 100 mmol) was dissolved in 50 mL of ethanol (solution C). Solution A was dropped into solution B slowly and heated with stirring at 50 °C for 3 h (solution D). Then, solution C was dropped into solution D and heated with stirring for another 3 h. Ethanol was distilled off under vacuum at 45 °C. The obtained gel was then re-dissolved in water to obtain a 1.0 M Ti^{4+}/Li^{+}-citrate chelate (TLCC) solution.

Synthesis of Resol Precursor

Resol was prepared in a base-catalyzed process from phenol and formaldehyde.[2] For a typical preparation, phenol (0.80 g, 8.5 mmol) was melted at 45 °C in a round-bottom flask and then 20% NaOH aqueous solution (0.17 g, 8.5 mmol) was added slowly over 5 min under stirring. Formalin (37 wt %, 1.45 g) containing formaldehyde (17.8 mmol) was dropped into this solution and the reaction mixture was further stirred at 70 °C for 1 h. After cooling the mixture to room temperature, the pH value was adjusted to neutral (~7.0) using dilute HCl solution (2.0 M). Water in the resol was removed by vacuum evaporation at 50 °C. The final product was dissolved in ethanol (20 wt %) before use.

Synthesis of Ultradispersed and Ultrasmall $LiTiO_2$ Nanocrystals/Ordered

Mesoporous Carbon Composites. The ultradispersed and ultrasmall $LiTiO_2$ nanocrystals/ordered mesoporous carbon ($LiTiO_2$-OMC) composites was synthesized *via* the molecular chelate strategy. In a typical synthesis, 1.5 g of triblock copolymer Pluronic F127, 5.0 g of the resol solution, and 2.0 mL of 1.0 M TLCC solution were dissolved in an ethanol/water mixture (16 mL, 4:1 v/v). After stirring for 2 h at room temperature, the obtained light yellow homogenous solution was poured into Petri dishes to evaporate the solvents at 40 °C for 8 h, followed by sequential solidifying the samples at 100 °C for 20 h in an oven to remove the solvents completely, leading to the formation of the as-made F127/Ti^{4+}/Li^{+}/citrate composites (denoted as as-made sample). Then the as-made sample was pyrolyzed at 900 °C for 3 h with a ramp of 2 °C min^{-1} in N_2 atmospheres, resulting in the $LiTiO_2$-OMC. In addition, $LiTiO_2$-OMC with different $LiTiO_2$ ratio (denoted as *X*-$LiTiO_2$-OMC, wherein *X* represents the $LiTiO_2$ percentage in the composites) were synthesized by adjusting the additional amount of TLCC precursor.

Synthesis of the S-based Cathodes. The S-based cathodes were prepared *via* the melt-diffusion method. Typically, 30 mg sulfur powder was mixed with 20 mg *X*-$LiTiO_2$-OMC with heat treatment at 155 °C for 12 h in a sealed glass vessel under Ar atmosphere to gain S/*X*-$LiTiO_2$-OMC cathode.

Characterization. Small-angle X-ray scattering (SAXS) measurements were taken on a Nanostar U small-angle X-ray scattering system (Bruker, Germany) using Cu Kα radiation (40 kV, 35 mA). X-ray diffraction (XRD) patterns were obtained from a Bruker D4 diffractometer within 2θ range from 10 to 90°, by using a Cu Kα radiation source (40 kV, 40 mA). Nitrogen adsorption−desorption isotherms were measured at 77 K with a Micromeritics Tristar 2420 analyzer (USA). Before measurements, the samples were degassed in a vacuum at 190 °C for 10 h. The Brunauer−Emmett−Teller (BET) method was utilized to calculate the specific surface areas. By using the Barrett−Joyner−Halenda (BJH) model, the pore volumes and pore size distributions were derived from the adsorption branches of isotherms, and the total pore volumes were estimated from the adsorbed amount at a relative pressure P/P_0 of 0.992. Transmission electron microscopy (TEM) measurements were conducted on o JEM-

2100 F microscope (JEOL, Japan) operated at 200 kV. The samples for the TEM measurements were suspended in ethanol and supported onto a holey carbon film on a Cu grid. Field-emission scanning electron microscopy (FESEM) images were taken on a Hitachi S-4800 microscope. The dried samples were directly used for the observation without any treatment. X-ray photoelectron spectroscopy (XPS) was recorded on an AXIS ULTRA DLD XPS System with MONO Al source (Shimadzu Corp). Photoelectron spectrometer was recorded by using monochromatic Al KR radiation under vacuum at 5×10^{-9} Pa. All calibrations were referenced to the surface adventitious carbon (C1s = 284.6 eV). Elemental analysis experiments were carried out using an Elementar Vario EL III microanalyzer. Raman spectra were recorded with a Dilor LabRam-1 B microscopic Raman spectrometer, using a He−Ne laser with an excitation wavelength of 632.8 nm.

Cell Assembly and Electrochemical Measurements. 2032-type coin cells were used to test the electrochemical performance of cathode materials with lithium metal as reference electrodes and Celgard 2400 membrane as the separator. Cathode materials were composed of 80% active materials, 10% polyvinylidenedifluoride (PVDF) and 10% carbon black. The electrolyte was composed of 1 M lithium bis(trifluoromethanesulfonyl)imide (LiTFSI) in a solvent mixture of 1,3-dioxolane (DOL) and dimethoxymethane (DME) (1:1 in volume) with 2 wt% $LiNO_3$ as an additive. The areal S loading was about 1.5 mg cm^{-2}. All the capacity values were calculated based on the mass of S. The galvanostatic electrochemical measurements were carried out in the Neware BTS-610 instrument with the voltage window of 1.6-2.8 V. The cyclic voltammetry (CV) test was performed in a scan rate of 0.2 mV s^{-1} and the electrochemical impedance spectrum (EIS) measurements were obtained in the frequency range of 100 kHz to 0.01 Hz on the CHI 660D workstation. Electrochemical impedance spectroscopy (EIS) spectra were collected from 100 kHz to 100 MHz at room temperature (25 °C).

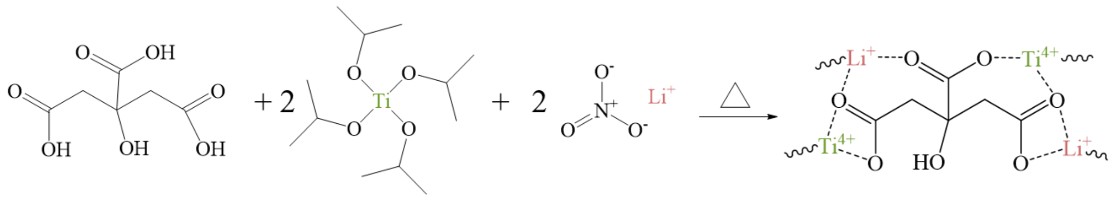

Figure S1. Illustration of the preparation process of stoichiometric Ti^{4+}/Li^{+}/citrate chelate (TLCC; Ti^{4+}: Li^{+} = 1:1).

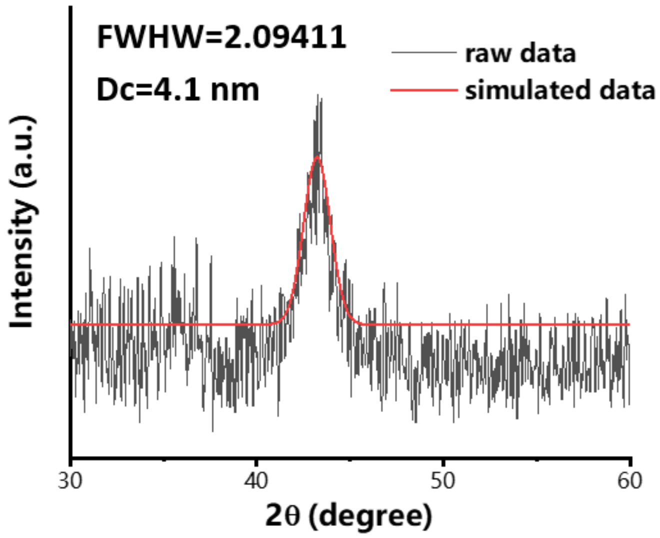

Figure S2: The Gaussian fitting of the XRD pattern of the 30%-$LiTiO_2$-OMC to calculate the crystalline size of $LiTiO_2$ nanocrystals based on Debye-Scherrer equation.

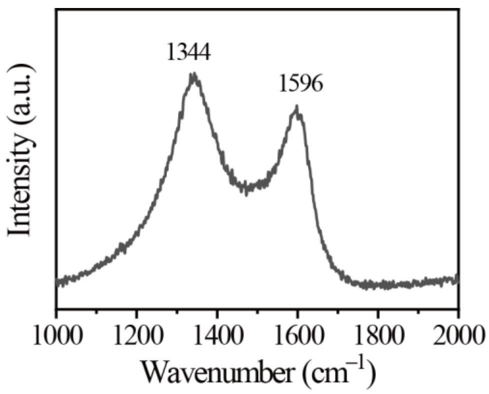

Figure S3. Raman spectrum of the 33%-$LiTiO_2$-OMC composites prepared by the chelation-mediated multicomponent co-assembly strategy after pyrolysis at 900 °C in N_2.

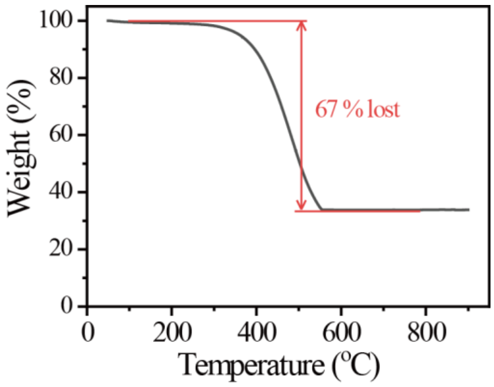

Figure S4. The TGA curve of the 33%-$LiTiO_2$-OMC composites prepared by the chelation-mediated multicomponent co-assembly strategy after pyrolysis at 900 °C in N_2 with a heating rate of 5 °C/min from 50 to 900 °C in air atmosphere. Approximately 67% weight loss is observed between 200 and 600 °C, which is attributed to the decomposition of carbon species in the composites.

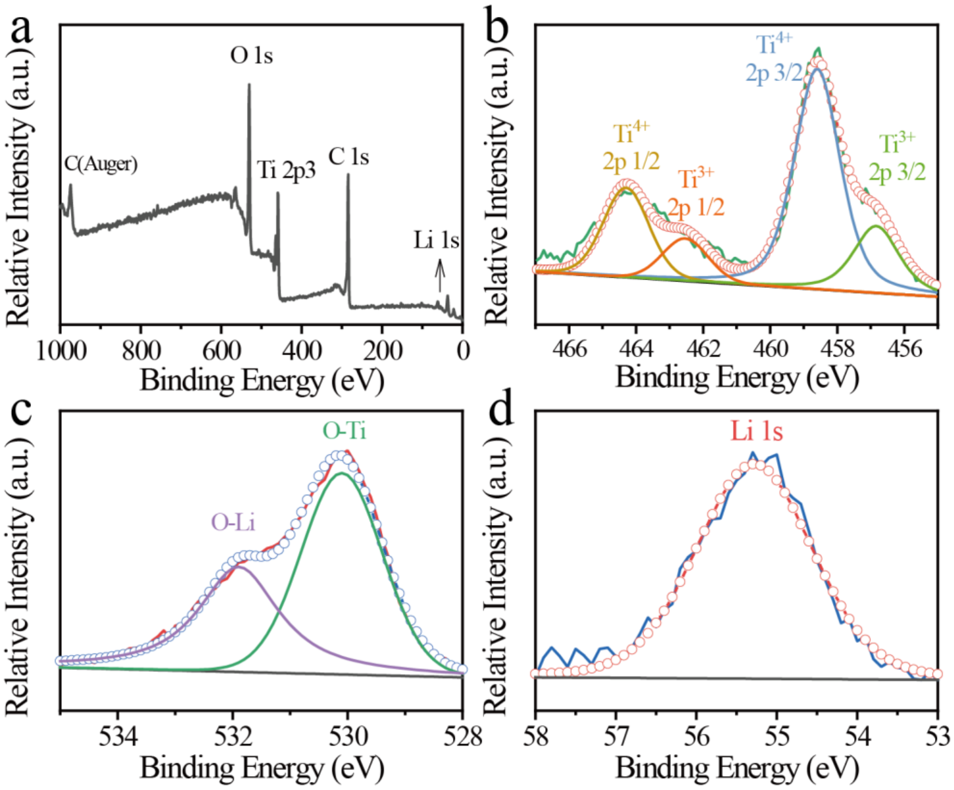

Figure S5. (a) The XPS survey spectrum and (b-d) XPS core-level spectra of Ti 2p, O 1s, and Li 1s of the 33%-$LiTiO_2$-OMC composites prepared by the chelation-mediated multicomponent co-assembly strategy after pyrolysis at 900 °C in N_2.

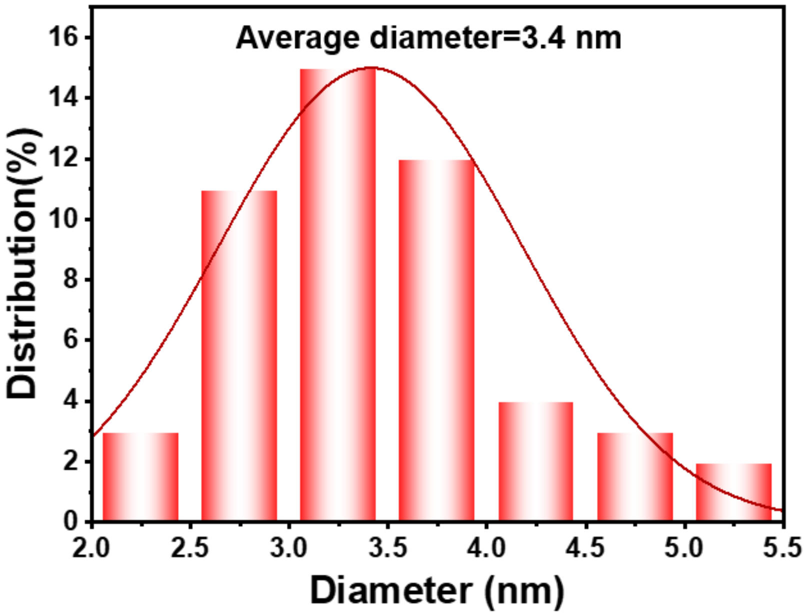

Figure S6: The diameter distribution histogram of $LiTiO_2$ nanocrystals in the 33%-$LiTiO_2$-OMC composite.

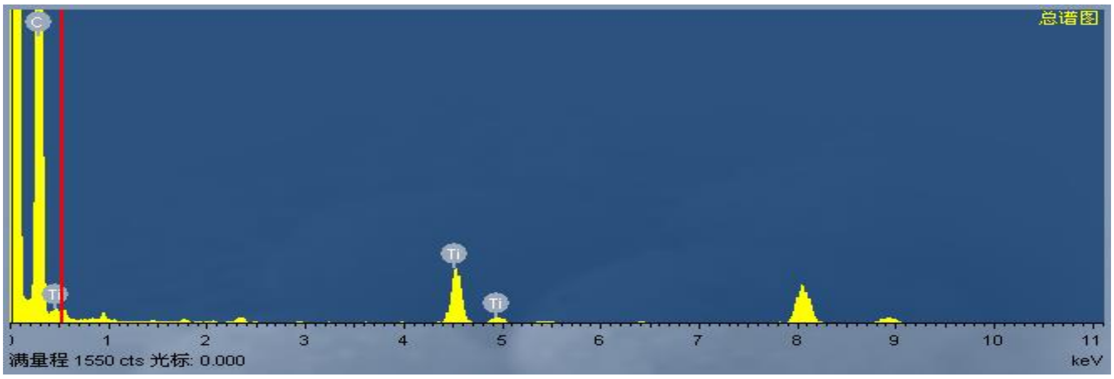

Figure S7. The EDX spectrum of the 33%-$LiTiO_2$-OMC composites prepared by the chelation-mediated multicomponent co-assembly strategy after pyrolysis at 900 °C in N_2.

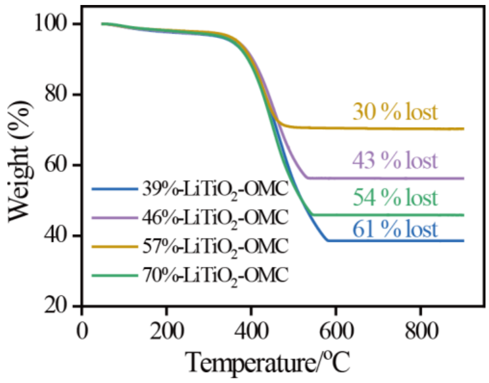

Figure S8. The TGA curve of the 39%-$LiTiO_2$-OMC, 46%-$LiTiO_2$-OMC, 57%-$LiTiO_2$-OMC, and 70%-$LiTiO_2$-OMC composites prepared by the chelation-mediated multicomponent co-assembly strategy after pyrolysis at 900 °C in N_2 with a heating rate of 5 °C/min from 50 to 900 °C in air atmosphere.

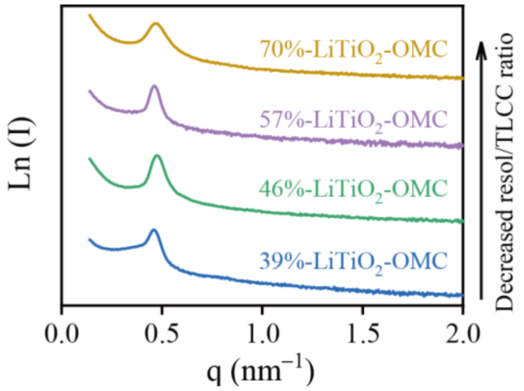

Figure S9. SAXS patterns of the as-made samples prepared by the chelation-mediated multicomponent co-assembly strategy by changing the mass ratio of resol/TLCC precursors.

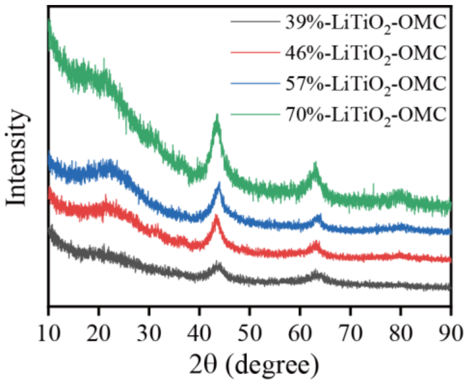

Figure S10. XRD patterns of the 39%-$LiTiO_2$-OMC, 46%-$LiTiO_2$-OMC, 57%-$LiTiO_2$-OMC, and 70%-$LiTiO_2$-OMC composites prepared by the chelation-mediated multicomponent co-assembly strategy after pyrolysis at 900 °C in N_2.

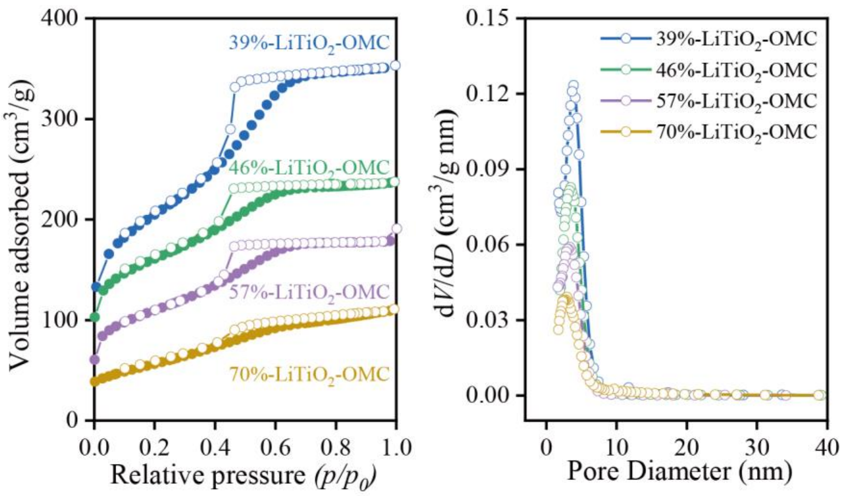

Figure S11. (a) N_2 sorption isotherms and (d) pore size distribution curves of the 39%-$LiTiO_2$-OMC, 46%-$LiTiO_2$-OMC, 57%-$LiTiO_2$-OMC, and 70%-$LiTiO_2$-OMC composites prepared by the chelation-mediated multicomponent co-assembly strategy after pyrolysis at 900 °C in N_2, respectively. The surface areas of each sample are 805, 536, 426 and 195 $m^2\ g^{-1}$, respectively. The pore volumes are 0.55, 0.37, 0.26, and 0.13 $cm^3\ g^{-1}$, respectively. The average pore sizes are calculated to be 3.8, 3.5, 3.3, and 3.0 nm, respectively.

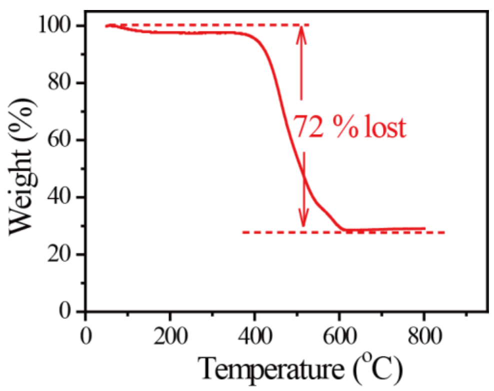

Figure S12. The TGA curve of the 28%-TiO_2/lithium titanate composites obtained after pyrolysis at 900 °C prepared by using TIPO and $LiNO_3$ as precursors with a heating rate of 5 °C/min from 50 to 900 °C in air atmosphere. Approximately 72% weight loss is observed between 200 and 600 °C, which is attributed to the decomposition of carbon species in the composites.

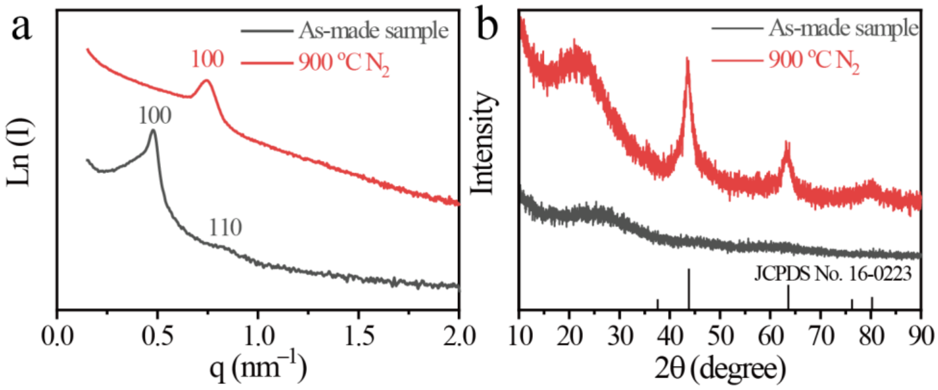

Figure S13. (a) SAXS and (b) XRD patterns of the as-made sample and the TiO_2/lithium titanate composites obtained after pyrolysis at 900 °C prepared by using TIPO and $LiNO_3$ as precursors.

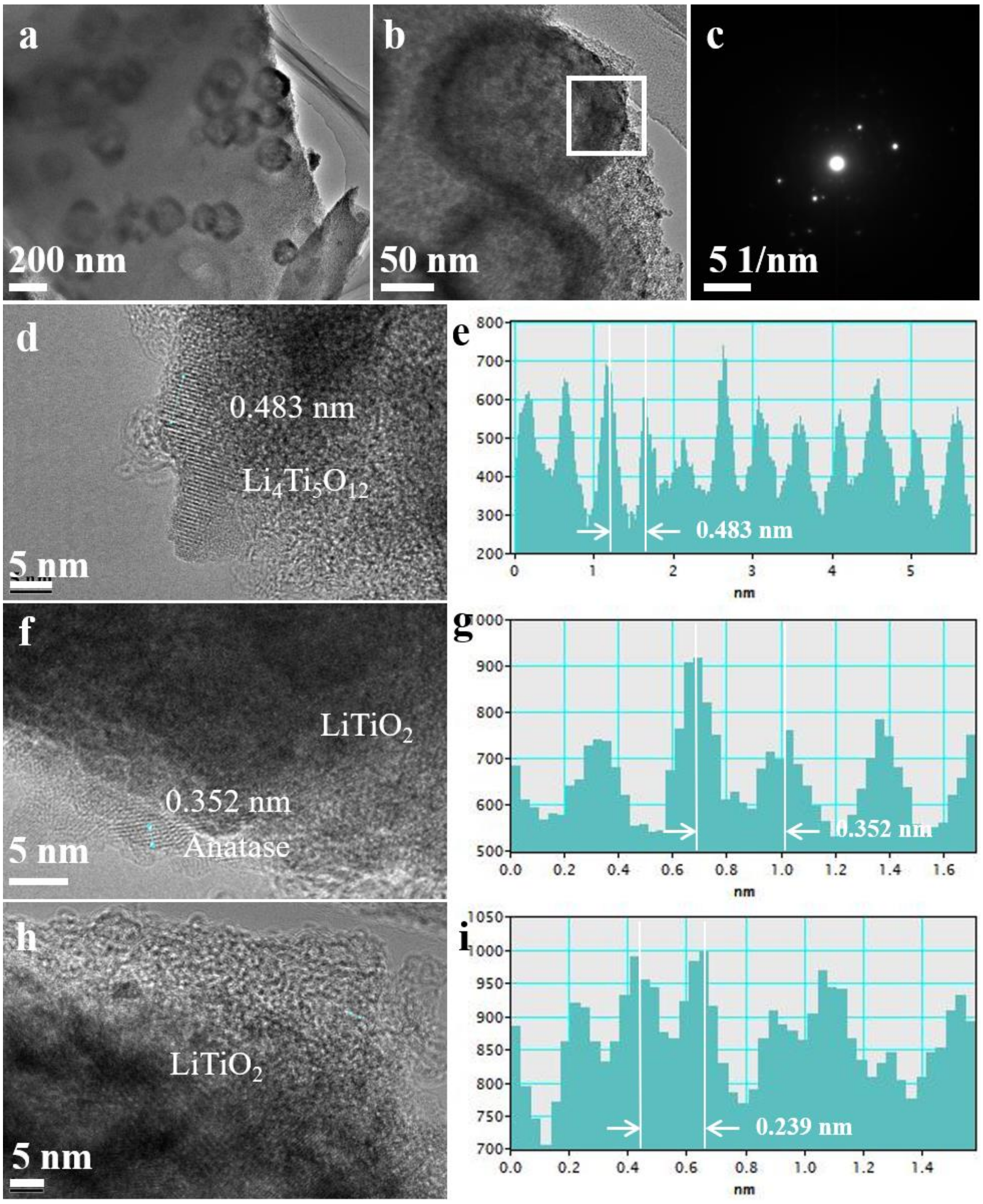

Figure S14. (a, b) TEM images and (c) corresponding SAED pattern of the TiO_2/lithium titanate composites obtained after pyrolysis at 900 °C prepared by using TIPO and $LiNO_3$ as precursors. (d, f, h) HRTEM images and (e, g, i) corresponding crystal lattice distance plots taken from the different part of the hollow particle from panel b.

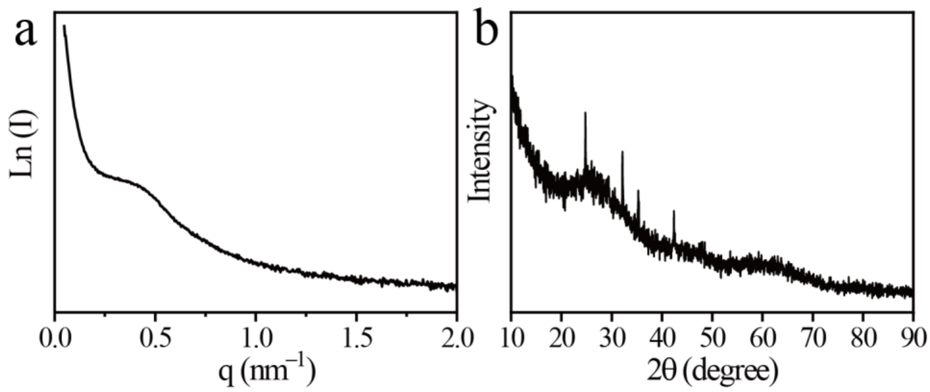

Figure S15. (a) SAXS and (b) XRD patterns of the as-made sample prepared by using TIPO and $LiNO_3$ as precursors with a high ratio of TIPO/$LiNO_3$ precursors.

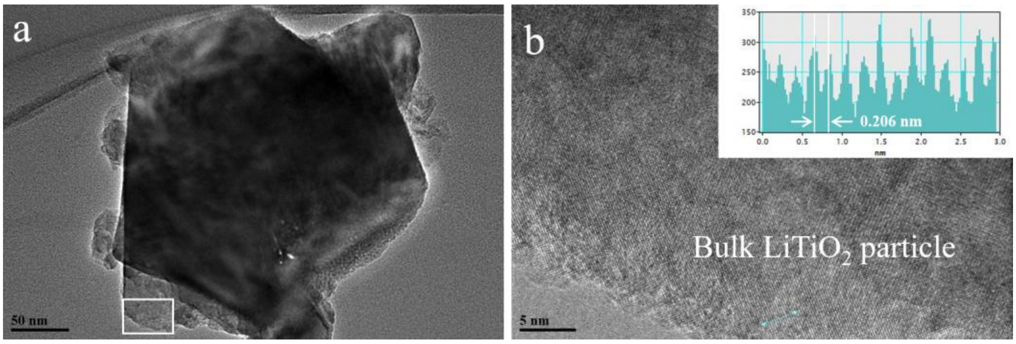

Figure S16. (a) TEM image and (b) HRTEM image of the sample obtained after pyrolysis at 900 °C in N_2 prepared by using TIPO and $LiNO_3$ as precursors with a high ratio of TIPO/$LiNO_3$ precursors. The inset in panel b is the corresponding crystal lattice distance plots of the crystal.

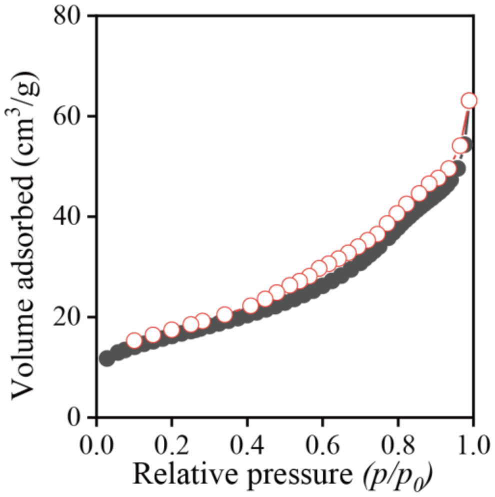

Figure S17. Nitrogen-sorption isotherms of the S/33%-$LiTiO_2$-OMC composites electrode.

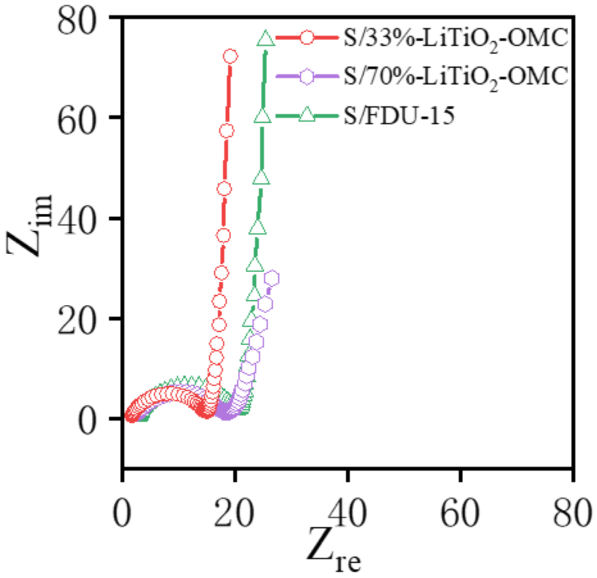

Figure S18. Nyquist plots of the S/33%-$LiTiO_2$-OMC composites, S/70%-$LiTiO_2$-OMC composites, and S/FDU-15 at room temperature (25 °C), respectively.

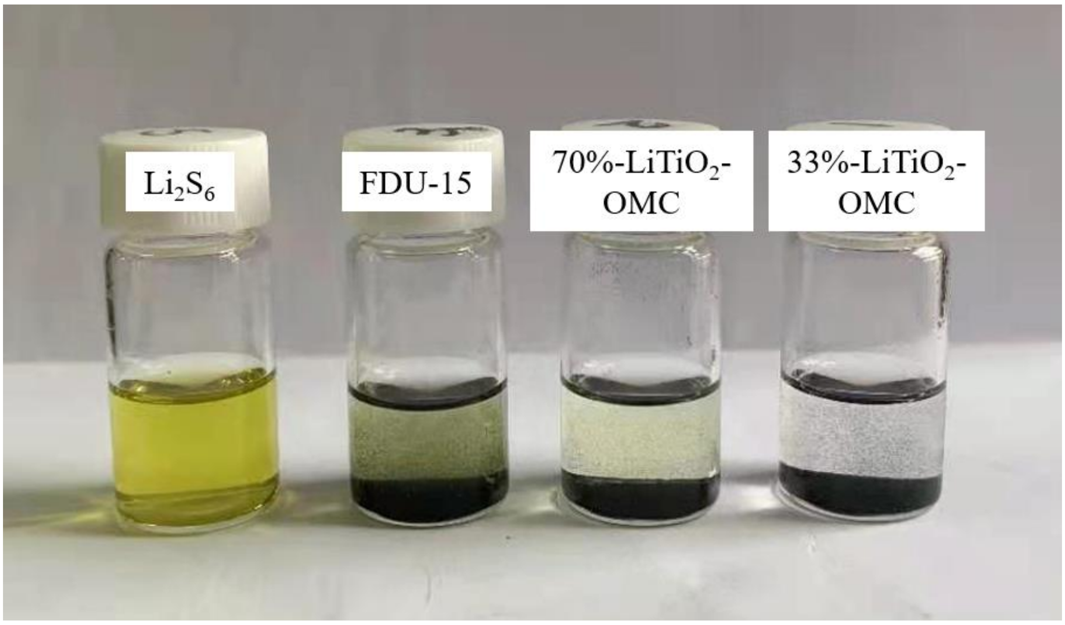

Figure S19. Optical photograph of the Li_2S_6 solutions in dimethoxyethane/dioxolane with the immersion of FDU-15, 70%-LiTiO$_2$-OMC composites, and 30%-LiTiO$_2$-OMC composites, respectively.

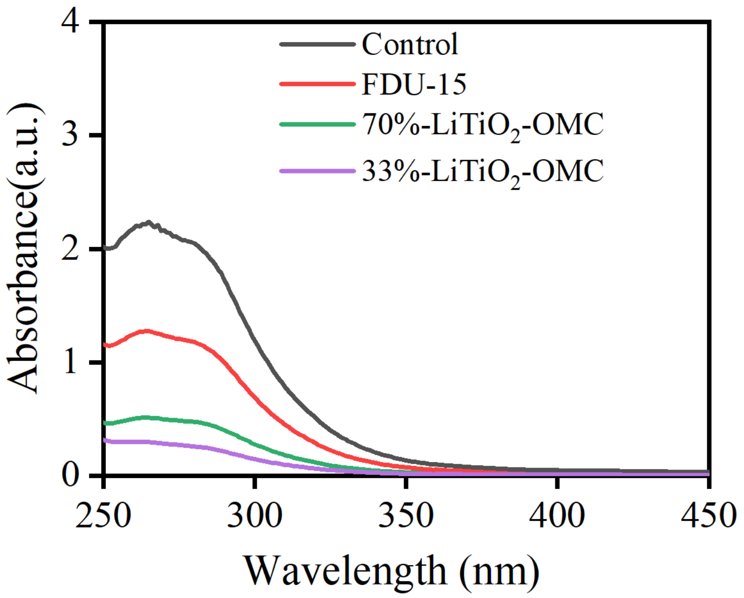

Figure S20. The UV-Vis spectra of different samples control, FDU-15, 70%-$LiTiO_2$-OMC composites, and 33%-$LiTiO_2$-OMC composites after soaking in the Li_2S_6 solution for 4 h, respectively.

Table S1. Structural and Textural Properties of the X-$LiTiO_2$-OMC

Sample	a_0 (nm) [a]	S_{BET} (m^2/g)	V (cm^3g^{-1}) [b]	D_p (nm) [c]	D_c (nm) [d]	$LiTiO_2$ (wt%) [e]
33%-$LiTiO_2$-OMC	8.8	916	0.62	4.1	4.0	33
39%-$LiTiO_2$-OMC	9.7	805	0.55	3.8	4.2	39
46%-$LiTiO_2$-OMC	9.3	536	0.37	3.5	4.5	46
57%-$LiTiO_2$-OMC	8.9	426	0.26	3.3	4.9	57
70%-$LiTiO_2$-	8.7	195	0.13	3.0	5.3	70

OMC						

[a] Cell parameter calculated from SAXS patterns. [b] Total pore volumes estimated based on the volume adsorbed at P/P0 of ~0.995. [c] Pore sizes derived from the adsorption branches of the isotherms by using the BJH method. [d] Average crystal size estimated by the Scherrer equation from XRD patterns. [e] $LiTiO_2$ weight percentage obtained by TGA curves.

Table S2: Comparison of performance of the 33%-$LiTiO_2$-OMC cathode with relevant literature with similar sulfur loading

Cathode	Sulfur loading (wt%)	Initial capacity (mAh/g)	Cycle number (n)	Retention capacity (mAh/g) and ratio (%)	Reference
30%-$LiTiO_2$-OMC	60	1131 (0.5 C)	100 (0.5 C)	966 (85%)	This work
Bronze TiO_2 Sheet	61	696 (0.5 C)	100 (0.2 C)	572 (66%)	*J. Energy Chem.* **2020**, 48, 259
MMC/TiO_2	66	795 (0.5 C)	60 (0.2 C)	931 (65.6%)	*ACS Appl. Energy Mater.* **2022**, 5, 2573
TiO_2-air	62	689 (0.5 C)	100 (0.2 C)	900 (61%)	*J. Mater. Chem. A* **2019**, 7, 10346
TiO_2/$BaTiO_3$ junction	62	898 (0.5 C)	500 (0.5 C)	541 (60%)	*Chem. Commun.* **2018**, 54, 12250
Tubular TiO_2/RGO	60	1200 (0.5 C)	200 (0.5 C)	630 (52.5%)	*Carbon*, **2018**, 128, 63

TiO_2 nanowire array	--	800 (0.5 C)	100 (0.5 C)	525 (65.6%)	*Electrochim. Acta*, **2018**, 264, 20

References

1. Wang, C.; Wan, X.; Duan, L.; Zeng, P.; Liu, L.; Guo, D.; Xia, Y.; Elzatahry, A. A.; Li, W.; Zhao, D., Molecular Design Strategy for Ordered Mesoporous Stoichiometric Metal Oxide. *Angew. Chem. Int. Ed.* **2019,** *58* (44), 15863-15868.

2. Meng, Y.; Gu, D.; Zhang, F.; Shi, Y.; Yang, H.; Li, Z.; Yu, C.; Tu, B.; Zhao, D., Ordered mesoporous polymers and homologous carbon frameworks: amphiphilic surfactant templating and direct transformation. *Angew. Chem. Int. Ed.* **2005,** *44* (43), 7053-9.
